# Contrasting altitudinal variation of alpine plant communities along the Swedish mountains

**DOI:** 10.1002/ece3.6237

**Published:** 2020-04-16

**Authors:** Johannes Måsviken, Fredrik Dalerum, Sara A. O. Cousins

**Affiliations:** ^1^ Department of Zoology Stockholm University Stockholm Sweden; ^2^ Department of Bioinformatics & Genetics Swedish Museum of Natural History Stockholm Sweden; ^3^ Centre for Palaeogenetics Stockholm Sweden; ^4^ Research Unit of Biodiversity (UMIB, UO‐CSIC‐PA) University of Oviedo Mieres Spain; ^5^ Department of Zoology and Entomology Mammal Research Institute University of Pretoria Hatfield South Africa; ^6^ Biogeography & Geomatics Department of Physical Geography Stockholm University Stockholm Sweden

**Keywords:** altitudinal gradient, community structure, elevation, latitude, mountain ecology, taxonomic diversity

## Abstract

Changes in abiotic factors along altitudinal and latitudinal gradients cause powerful environmental gradients. The topography of alpine areas generates environmental gradients over short distances, and alpine areas are expected to experience greater temperature increase compared to the global average. In this study, we investigate alpha, beta, and gamma diversity, as well as community structure, of vascular plant communities along altitudinal gradients at three latitudes in the Swedish mountains. Species richness and evenness decreased with altitude, but the patterns within the altitudinal gradient varied between sites, including a sudden decrease at high altitude, a monotonic decrease, and a unimodal pattern. However, we did not observe a decline in beta diversity with altitude at all sites, and plant communities at all sites were spatially nested according to some other factors than altitude, such as the availability of water or microtopographic position. Moreover, the observed diversity patterns did not follow the latitudinal gradient. We observed a spatial modularity according to altitude, which was consistent across sites. Our results suggest strong influences of site‐specific factors on plant community composition and that such factors partly may override effects from altitudinal and latitudinal environmental variation. Spatial variation of the observed vascular plant communities appears to have been caused by a combination of processes at multiple spatial scales.

## Graphical abstract

We studied the altitude variation of alpine plant communities in the Swedish mountains. We found that species richness and evenness decreased with altitude but the patterns within the altitude gradient varied between sites, including a sudden decrease at high altitude, a monotonic decrease, and a unimodal pattern.

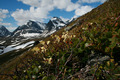

## 1 INTRODUCTION

Together with the large‐scale latitudinal gradient in species richness (Currie, 1991), variation in species communities across altitude remains one of the most widely studied environmental gradients affecting species communities (Lomolino, 2001; Whittaker, 1960). Mountain areas with their steep topography generate climatic gradients where temperature typically decreases by 0.5–0.7°C per 100 m in elevation gain (Beniston, Diaz, & Bradley, 1997; Grabherr, Gottfried, Gruber, & Pauli, 1995). These altitudinal gradients in climatic conditions, combined with altitudinal variation in other environmental factors such as moisture and general available area, can be major factors driving community structuring and spatial variation in species richness (Barry, 2008; Körner, 2000; Whittaker, 1960). However, despite general declines in diversity and species richness with altitude, the patterns with which species communities varies along altitudinal gradients are not uniform (Rahbek, 1995). Although the importance of spatial scale for altitudinal variation in species communities has been recognized (Rahbek, 2005), we still have limited understanding of what scales different processes act and interact in shaping altitudinal gradients in species diversity.

Combined, the two‐dimensional gradient of altitude and latitude, in which climate and ultimately net available energy vary in predictable ways along each dimension, albeit at different spatial scales, provides a powerful framework for scale‐dependent evaluations of community structuring (Jump, Mátyás, & Peñuelas, 2009; Körner, 2003; Maček, Vodnik, Pfanz, Low‐Décarie, & Dumbrell, 2016). Alpine vegetation, that is, vegetation found above the tree line (Körner, 2000), is generally structured into three vegetation zones which have a vertical extent of a couple of hundred elevation meters, although their extent depends on the level of maritime influences on local climate (Körner, 2003; Rydin, Snoeijs, & Diekmann, 1999). However, the altitude at which alpine vegetation occurs also varies with latitude. The highest tree line is found close to the equator, at approximately 4,800 m.a.s.l., from which the alpine zone is found at successively lower altitudes toward the poles (Hoch & Körner, 2005; Körner, 2003). Understanding how the spatial variation in community structuring of alpine vegetation varies with latitude is of particular relevance in the face of the ongoing climate change, since both alpine and northern environments are predicted to experience the biggest ecological impacts from global warming (Bekryaev, Polyakov, & Alexeev, 2010; Beniston et al., 1997; IPCC , 2013; Pepin et al., 2015).

Biodiversity is frequently divided into alpha, beta, and gamma components (Whittaker, 1960), where alpha diversity describes the diversity within locations, gamma diversity describes the diversity within landscapes, and beta diversity describes the difference in diversity between locations within landscapes. Most research on biodiversity variation along altitudinal gradients has either focused on variation in alpha diversity (e.g., Rahbek, 1995) or on variation in beta diversity along the gradients (which has been used to quantify the gradient itself, Anderson et al., 2011). Along altitudinal gradients, species richness (a measure of taxonomic alpha diversity) most frequently displays a hump‐shaped pattern (Rahbek, 1995). This pattern has generally been attributed to productivity‐associated competitive exclusion at low altitudes and a productivity‐driven filtering of species communities at high altitudes (Huston, 1999). Less attention has been given to spatial variation in communities across altitude (Rahbek, 2005). Although beta diversity is highly influenced by the spatial scale over which it is measured, the dilution of communities at high altitudes is expected to give rise to a spatial homogenization and a subsequent altitude‐related decline in beta diversity (Naud et al., 2019). Such patterns have been observed, but do not appear to be uniform across all spatial scales (Naud et al., 2019). Alpha and beta diversity, and subsequently gamma diversity, is driven by primary productivity and increasing altitude decreases productivity. Thus, we would expect that spatial diversity components and their relationship would change along altitudinal gradients.

While the spatial diversity components proposed by Whittaker (1960) describe several important characteristics of spatial community variation, they do not elucidate all spatial patterns of community structuring (Dalerum, Vries, Pirk, & Cameron, 2017). In particular, spatial nestedness and modularity are important structures that are not captured by alpha, beta, and gamma diversity (Leibold & Mikkelson, 2002). Although rarely used in evaluation of altitudinal effects on diversity, nested and modular patterns are highly informative for the processes shaping potential altitudinal gradients in diversity. In spatially nested patterns, the most species poor locations are inhabited by species that also exists in more species‐rich ones, for example, generalists species (Almeida‐Neto, Frensel, & Ulrich, 2012; Thébault, 2013; Wright, Patterson, Mikkelson, Cutler, & Atmar, 1997). Modular spatial community structures, on the other hand, result from species turnover and consist of local communities consisting of unique combinations of species, for example, by specialists species. Modularity is a well‐established concept in nonspatial networks such as food webs and social networks (Newman, 2006), but are less investigated in biogeography partly because of previous lack of sufficient theoretical work and statistical methods (Hausdorf & Hennig, 2007; Thébault, 2013). Nested and modular structures are highly suitable to evaluate community transition along environmental gradients (Naud et al., 2019), where nested patterns suggest that communities at low diversity sites are maintained by generalist species. Modular patterns, on the other hand, suggest that each level of the gradients is inhabited by species specialized to its specific conditions. It should be noted that nested and modular structures are not mutually exclusive, for example, spatial nestedness can be found within spatial modules (Fortuna et al., 2010).

In this study, we investigate variation in alpha diversity, measured as species richness and species evenness, beta diversity, and spatial community structure of plant communities along altitudinal gradients at three sites at different latitudes in the Swedish mountains. While Naud et al. (2019) recently highlighted variation in altitudinal gradients of alpine vegetation within a single alpine landscape in northern Sweden, we here evaluate if large‐scale latitudinal contrasts in altitudinal gradients may override such landscape level variations. We focus on strict quantification of patterns of community variation without evaluating potential hypotheses for what drives these potential patterns, since we regard it appropriate to first identify patterns before rigorous tests are developed to evaluate their causes (Dalerum, 2012). Based on empirical observations, we expect a unimodal or monotonically decreasing alpha diversity (species richness and species evenness) with increasing altitude (Bruun et al., 2006; Naud et al., 2019; Rahbek, 1995, 2005). We also expect a decline in beta diversity with increasing altitude, due to a spatial homogenization of species poor communities at high altitudes. Furthermore, we expect both of these two patterns to be weaker at higher latitudes, due to lower primary productivity at lower altitudes. Finally, we aim to test whether altitudinal variation in plant community composition is driven by species turnover and subsequently by altitudinal specialists or by continuous species loss where high‐altitude communities consist of altitudinal generalists, as well as whether such patterns are influenced by latitude.

## 2 METHODS

### 2.1 Study area

The Swedish mountains form parts of the Scandes mountain range and have a north‐to‐south orientation with an extent of approximately 950 km. The main part of the Swedish mountains consists of Caledonian bedrock, formed ca 450 million years ago. The Caledonian bedrock mostly overthrust the older basal granitoid bedrock beneath, and forms a diverse mix of different layers of metamorphic and sedimentary bedrock. Botanically important calcareous bedrock occurs in patches and mainly in central Scandes (SGU, 2019; Sjörs, 1999). The upper tree line varies greatly with latitude and local climatic conditions along the mountain range, from ~600 m.a.s.l. in the north to 1,000–1,200 m.a.s.l. in the south (Odland, 2015). In Scandinavia, the alpine vegetation starts at the tree line and is divided into three different vegetation zones. The tree line is natural but defined by complex interactions between abiotic and biotic factors (Körner, 2003; Moen, Cairns, & Lafon, 2008). The low alpine zone ranges from the tree line up to where *Vaccinium myrtillus* ceases and is dominated by heath vegetation with dwarf shrubs and meadows with graminoids. The middle alpine zone is increasingly more dominated by grasses, sedges, and rushes, with fewer and lower postured dwarf shrubs and extends to where there is no continuous vascular plant cover left. The upper border of the middle alpine zone is often diffuse and dependent on substrate and microclimatic conditions such as the accumulation and longevity of snow beds. The high alpine zone is dominated by rocky open ground, and the vegetation is sparse with single individuals or small groups of vascular plants, while bryophytes and lichens can form small dense communities (Barry, 2008; Nilsson, 1991; Rydin et al., 1999). Generally, the extent of the alpine vegetation zones is narrower in the west as these areas are more dominated by oceanic climate with colder summers; thus, the upper limit of the alpine zones sinks westward in the Scandinavian mountains (Rydin et al., 1999). Dominant vertebrate herbivores are willow grouse (*Lagopus lagopus*) and rock ptarmigan (*Lagopus muta*), mountain hare (*Lepus timidus*), several species of microtine rodents, and at lower elevations also moose (A*lces alces*). All but the southernmost part of the Swedish mountains are subjected to substantial grazing by semidomestic reindeer (*Rangifer tarandus*) (Moen, 2008). The mountains are popular recreation areas with activities such as hiking during summer and skiing and snowmobiling during winter (Heberlein, Fredman, & Vuorio, 2002). However, areas above the tree line are almost exclusively without roads, with limited human infrastructure and no resident human settlements.

### 2.2 Study sites

**FIGURE 1:**
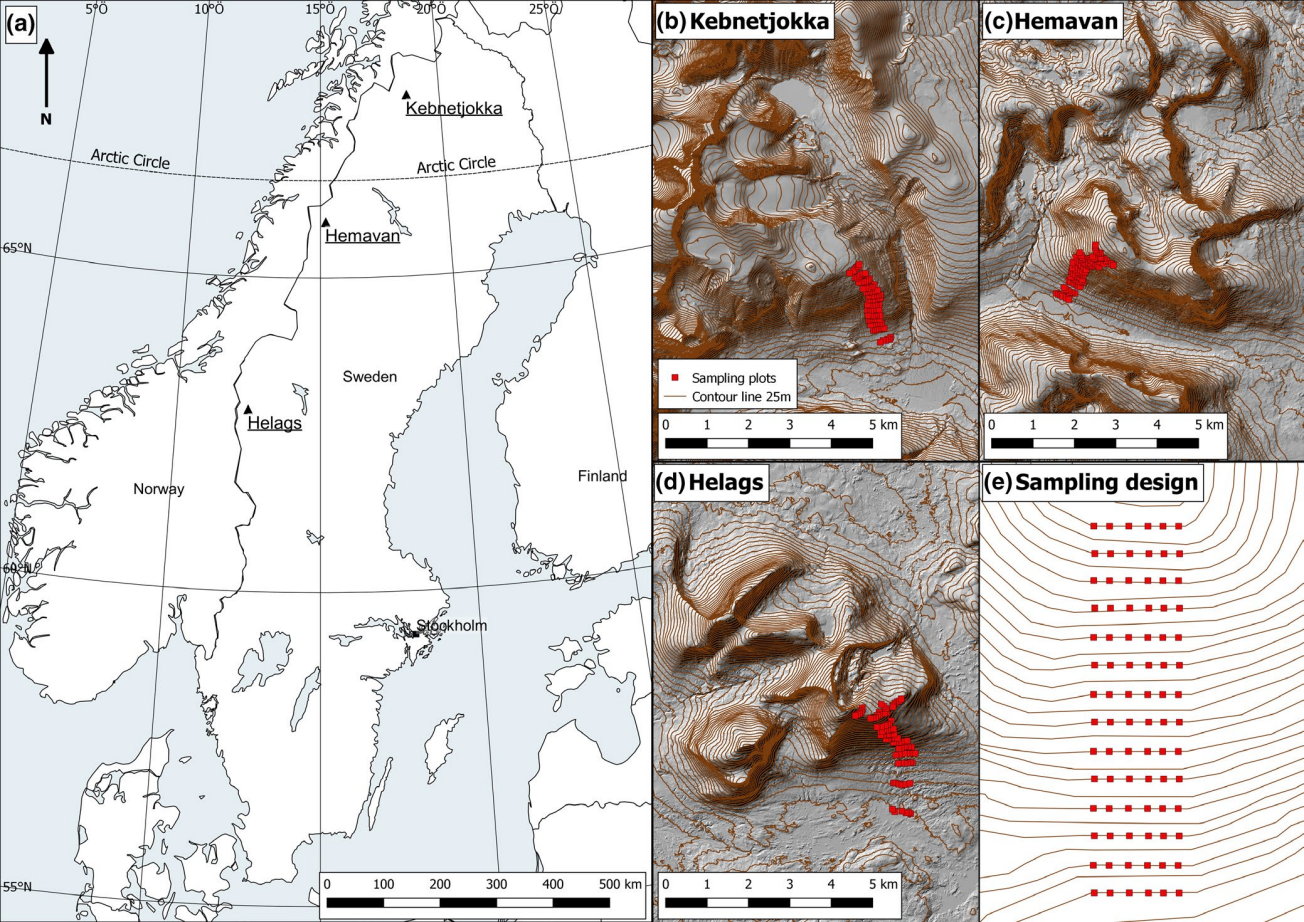
Positions of the three study sites in northwestern Sweden (a). The sampling plots, 25 m contour lines and a hillshade background derived of data from Lantmäteriet GDS—höjddata 2 m, at the northern site (b), central site (c), and southern site (d). A generalized layout of the sampling design, 14 horizontal transect each consisting of six 1 m^2^ plots (e)

We collected field data at three southern‐facing mountain sites: one in the northern (Kebnetjokka), one in the central (Hemavan), and one in the southern (Helags) part of the Swedish mountains (Figure 1 & Table 1). The sites abiotic conditions such as bedrock, solar radiation, slope, and aspect were compared using ArcMap and QGIS (Table 1; ESRI, 2015; QGIS Development Team, 2018). We focused the study on plant diversity along south‐facing altitudinal gradients, partly to allow for a certain topographic consistency, but also since south‐facing aspects have been shown to respond more strongly to climate variations under certain conditions (Danby & Hik, 2007). The sites were distributed from 62°53 to 67°52, and field sampling took place above the tree line at each site. The sites were dominated by alpine heath vegetation with dwarf shrubs and with increasing proportion of graminoids with higher altitude. The distances between the sites are 267 km between the northern and central and 360 km between the central and the southern site, respectively, covering a north–south gradient of over 550 km.

**TABLE 1:** Study site description with site name, location, coordinates, mountain altitude, altitude of closest treeline, sampled elevation range, bedrock conditions (SGU, 2019), and average monthly temperature and precipitation for the summer and winter months, respectively, for the period 1961–1990 (SMHI, 2018)

	Kebnetjokka	Hemavan	Helags
Site location	Northern site	Central site	Southern site
Coordinates	67°52N, 18°37E	65°53N, 15°12E	62°53N, 12°28E
Mountain altitude	1,766 m.a.s.l.	1,699 m.a.s.l.	1,797 m.a.s.l.
Altitude of closest treeline	670 m.a.s.l.	730 m.a.s.l.	910 m.a.s.l.
Sampled elevation range	700–1,350 m.a.s.l.	850–1,500 m.a.s.l.	950–1,600 m.a.s.l.
Bedrock conditions	Sandstone, meta‐arkose, marble, amphibolite	Meta‐graywacke, phyllite, schist, quartzite	Sandstone, amphibolite, paragneiss
Average monthly temperature			
Summer	5.0°C	10.2°C	7.5°C
Winter	−11.3°C	−11.2°C	−8.2°C
Average monthly precipitation			
Summer	63.8 mm	69.2 mm	69.0 mm
Winter	28.3 mm	66.2 mm	45.4 mm
Estimated solar radiation	726,015 WH/m^2^	793,687 WH/m^2^	845,892 WH/m^2^

Note

Estimated solar radiation as average value for a buffer zone of 50 m around all plots per site for the year 2016.

### 2.3 Data collection

Data collection was conducted between June 27 and July 17 in 2016 starting at the southern site and successively sampling toward the north. At each study site, we used a stratified random sampling design in which 14 transects, each at a fixed and consistent altitude, were placed along an altitudinal gradient. Transects were spaced 50 elevation meters apart and spanned a total of 650 elevation meters. The first transect was placed in the beginning of each mountain's southern slope, above the tree line (forest line) but below the tree species limit as defined by Körner (2003), and continued to the high alpine vegetation zone. Since the mountain slopes started at different altitudes, the sampled altitude range differed between sites (Table 1). Each transect consisted of six square sampling plots of 1 m^2^, spaced between 30 and 150 m apart, resulting in a total of 84 plots per site. To minimize the risk of biased results, we avoided areas in immediate proximity to waterways as well as recently snow‐covered areas. We also restricted plot placements to locations with at least one vascular plant. The elevation at each plot was measured using a handheld GPS, and the plots were aligned along each mountain slope.

Within each 1 m^2^ plot, all vascular plants were identified down to lowest possible taxonomic level using the flora by Krok, Almquist, Jonsell, and Jonsell (2013). To enable calculations of biodiversity metrics based on relative abundance, we counted the number of plant species in five smaller 20 × 20 cm^2^ within each sample plot, one in the middle and one in each corner. A species could thus get a relative abundance value from 0 to 5 in each plot. All taxonomic nomenclature follows the Swedish taxonomic database Dyntaxa (2020). Vascular plant specimens that could not be identified to species level were identified to taxonomic level of genus or in rare cases family (Tables A1, A2, A3).

### 2.4 Data analysis

Species richness was defined as the number of species or taxa identified within each 1 m^2^ sampling plot. Species evenness was calculated using the Shannon diversity index (Hill, 1973a; Shannon, 1948), based on the relative abundance value of identified species within each plot. We used a Euclidean distance to centroid based metric of beta diversity, which has been regarded as appropriate for evaluating turnover along environmental gradients (Anderson, 2006). We calculated this metric in two steps. First, we constructed a pair‐wise distance matrix for each site using Bray–Curtis dissimilarities based on binary presence–absences of species in each plot. These were converted into principal coordinate space, in which we calculated the Euclidian distance from each plot to the centroid of all six plots along the same transect. Hence, this value gives a relative beta diversity measurement at each altitude along the slope.

We used generalized linear models with a Poisson error structure and a log link to evaluate the effect of altitude on plant species richness, and linear models to evaluate the effect of altitude on species evenness and beta diversity. We fitted separate models for each site and community metric, all which contained altitude as a continuous predictor. Because we observed tendencies of nonlinear relationships between plant community metrics and altitude, we evaluated a series of models from a simple linear relationship to segmented regressions with up to three break points. Segmented regression has been suggested as a robust and practical alternative to nonlinear regression or more sophisticated nonlinear techniques such as generalized additive models (Muggeo, 2003). We used an algorithm was the number of break points were prespecified a priori, and the value of the breakpoints and the slope between them were estimated using a bootstrap algorithm (Muggeo, 2008; Wood, 2001). We used Akaike information criterion (AIC, Akaike, 1974) to evaluate which model provided the best fit to each site and plant community metric. Models within two AIC units were considered to have equal empirical support (Burnham & Anderson, 2002).

We calculated nestedness and modularity using binary matrices where each row represented a sample plot and each column a plant species. We evaluated nestedness and modularity for each site separately. We evaluated the nestedness of vascular plant community composition using the NODF algorithm, which calculates nestedness based on decreasing fill and paired overlap (Almeida‐Neto, Guimarães, Guimarães, Loyola, & Ulrich, 2008). The value of the NODF index ranges from 0 (indicating no nestedness) to 100, indicating perfect nestedness (Almeida‐Neto et al., 2008), but since the NODF algorithm is dependent on matrix fill, it is recommended that observed values are evaluated against appropriate null models (Ulrich, Almeida‐Neto, & Gotelli, 2009). We used Barber's (2007) *Q* statistic calculated using the iterative BRIM (Bipartite Recursively Induced Modules) algorithm to evaluate spatial modularity in community structuring. Barbers *Q* is a null model‐dependent statistic that expands Newman and Girvan's (2004) initial concept of modularity to bipartite interaction matrices (Fortunato & Hric, 2016).

To provide a heuristic evaluation if any nested or modular structure followed the altitudinal gradient, we evaluated two separate methods of sorting the matrices. First, we calculated the indices using a sorting of rows and columns that generates optimal nestedness and modularity. For nestedness, these matrices were constructed by sorting according to row (number of species per plot) and column (number of occurrences per species) totals (Ulrich et al., 2009), and for modularity, by sorting according to plot and species scores obtained by reciprocal averaging (Hill, 1973b). Reciprocal averaging is an indirect ordination technique that finds ordination scores that optimize the correlation between a set of species and the locations in which they are sampled, and is particularly useful for evaluating environmental gradients (Gauch, Whittaker, & Wentworth, 1977). We also sorted the columns in the plot/species matrices by altitude. For each matrix, we compared observed values of nestedness and modularity against expected values from 1,000 null models. We used a null model designed for describing plant communities along gradients (Jonsson, 2001). This null model preserved the average column (i.e., species) frequencies, and hence, also on average the same matrix fills as the observed data. All analyses were made in the R statistical environment (version 3.5.1, R Core Team, 2018, http://www.r‐project.org) and the contributed packages metacom (version 1.5.1, Dallas, 2014, 2018), perm (version 1.0‐0.0, Fay & Shaw, 2010), segmented (version 0.5‐3.0, Muggeo, 2008), and vegan (version 2.5‐4, Oksanen et al., 2018).

## 3 RESULTS

In total, we identified 79 vascular plant taxa of which 63 (82.3%) could be identified to the taxonomic level of species (Tables A1, A2, A3). The northern site was most species‐rich with a total of 64 identified taxa, compared to 46 for the central and 58 for the southern sites. The northern site also had a higher number of unique taxa (16 taxa), that is taxa that did not occur in the other sites, compared to the other two sites (central site 1; southern site 6). The highest number of species per plot was 18 (at 950 and 1,100 m.a.s.l.), 22 (at 850 m.a.s.l.), and 23 (at 1,200 m.a.s.l.), for the northern, central, and southern site, respectively, and the lowest number was 1 (at 1,350 m.a.s.l.), 1 (at 1,450 m.a.s.l.), and 2 (at 1,500 and 1,600 m.a.s.l.).

**FIGURE 2:**
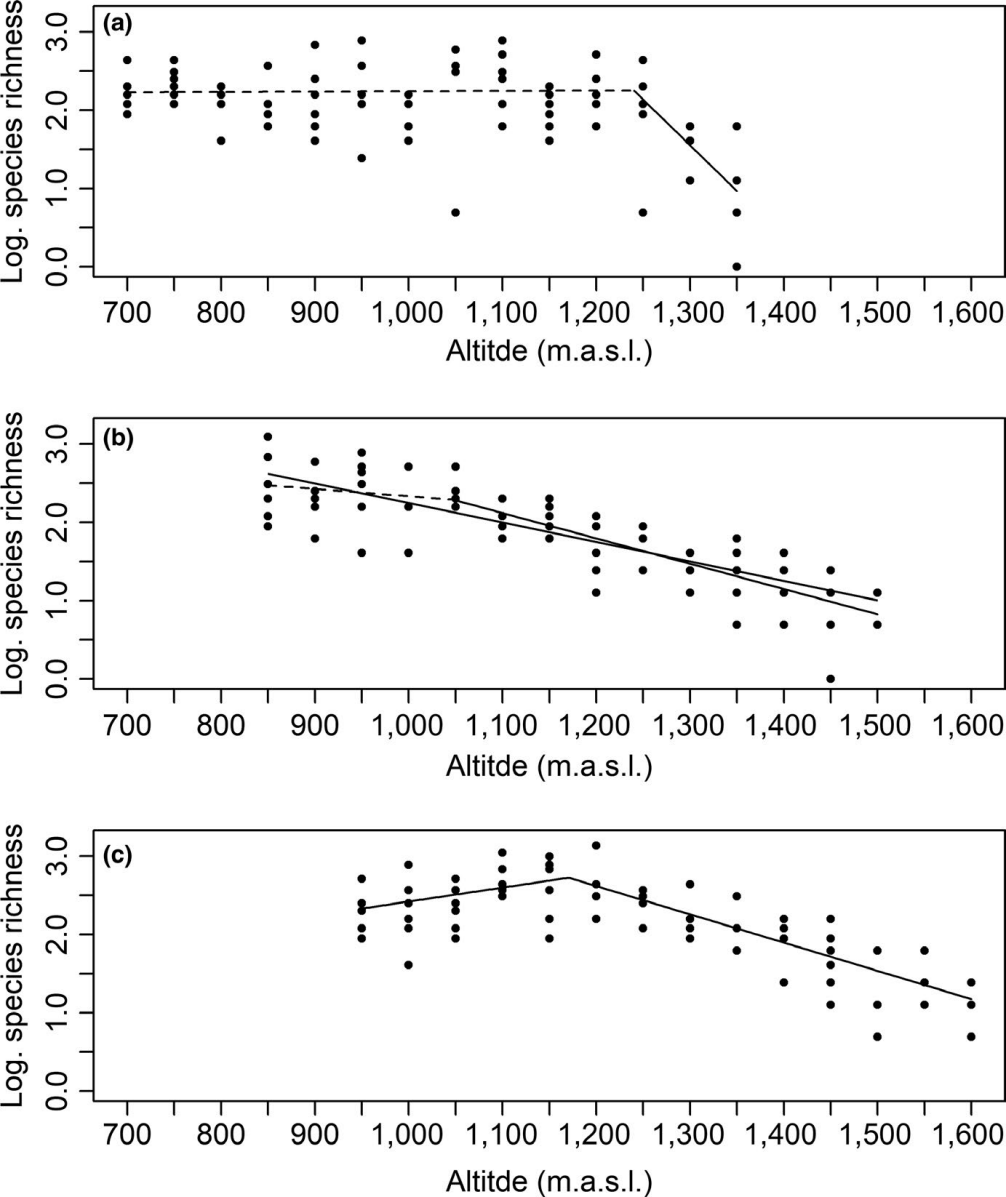
Relationships between vascular plant species richness and altitude at three study sites along the Swedish mountains: (a) Kebnetjokka, a northern site, (b) Hemavan, a central site, and (c) Helags, a southern site. Each data point reflects the natural logarithm of the species richness in 1 m^2^ plots. The lines show the general linear model and segmented linear models which provided the best fit for the data, and dashed lines show nonsignificant linear relationship

For species richness, segmented models with one breakpoint had the highest empirical support for the northern and southern sites, whereas both this model and an unsegmented linear model had equal empirical support for the central site (Table 2). At the northern site, species richness did not vary significantly with altitude below 1,240 (±1 *SE* = 14.85) m.a.s.l. (*β* = 4.20 × 10^–5^, *SE* = 2.54 × 10^–4^, *p* = .869), whereas species richness decreased significantly above 1,240 m.a.s.l. (*β* = −1.17 × 10^–2^, *SE* = 2.73 × 10^–3^, *p* < .001, Figure 2a). A similar pattern was observed at the central site, with no significant altitudinal variation in species richness until 1,047 (±76.40) m.a.s.l. (*β* = −9.28 × 10^–4^, *SE* = 1.10 × 10^–3^, *p* = .401), and a significant decline above this altitude (*β* = −3.23 × 10^–3^, *SE* = 4.17 × 10^–4^, *p* < .001). An equally supported model for the central site described a continuous altitudinal decline in species richness (*β* = −2.48 × 10^–3^, *SE* = 2.21 × 10^–4^, *p* < .001, Figure 2b). At the southern site, species richness increased significantly with altitude up to 1,171 (±24.19) m.a.s.l. (*β* = 1.78 × 10^–3^, *SE* = 7.37 × 10^–4^, *p* = .016). Above this, there was an altitudinal decline in species richness (*β* = −3.61 × 10^–3^, *SE* = 4.13 × 10^–4^, *p* < .001) (Figure 2c).

**TABLE 2:** Delta AIC scores, that is, the difference between a given model and the model with the lowest AIC value, for models evaluating the relationships between species richness, species evenness, and beta diversity and altitude

Model	Δ AIC		
	Kebnetjokka	Hemavan	Helags
Species richness			
No breakpoints	31.32	1.96	41.35
1 breakpoint	0.00	0.00	0.00
2 breakpoints	2.82	3.25	3.37
3 breakpoints	6.88	7.22	3.79
Species evenness			
No breakpoints	36.07	5.08	32.12
1 breakpoint	0.00	2.96	0.00
2 breakpoints	2.66	0.00	3.09
3 breakpoints	4.48	5.27	4.71
Beta diversity			
No breakpoints	3.37	0.00	0.00
1 breakpoint	0.00	0.59	3.36
2 breakpoints	2.80	2.63	4.90
3 breakpoints	6.54	5.92	6.33

Note

The sites Kebnetjokka, Hemavan, and Helags are situated along the Swedish mountains from north to south.

**FIGURE 3:**
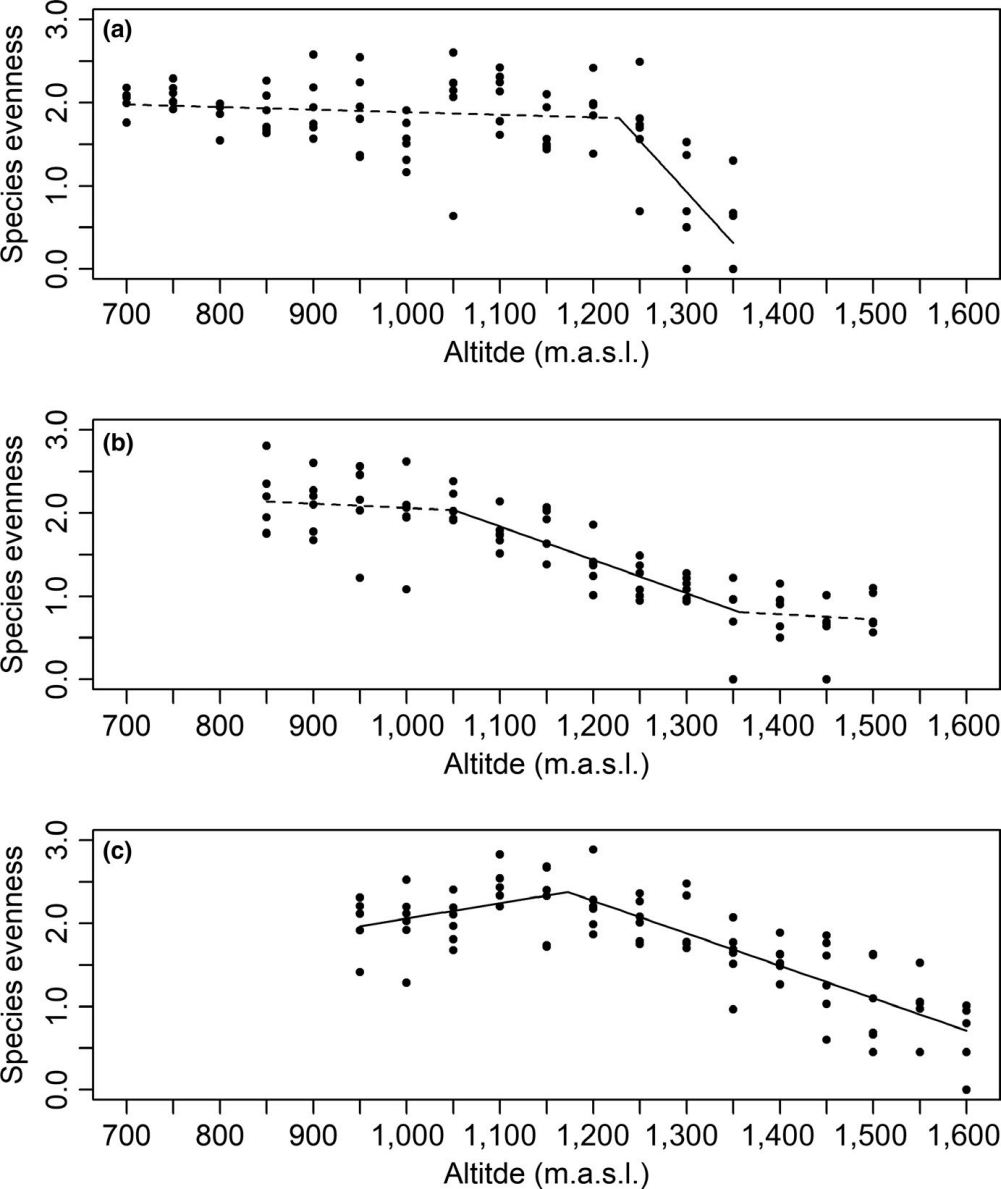
Relationships between vascular plant species evenness and altitude at three study sites along the Swedish mountains: (a) Kebnetjokka, a northern site, (b) Hemavan, a central site, and (c) Helags, a southern site. Each point reflects the Shannon index of species evenness for 1 m^2^ plots, which were calculated using the presence and absence of plant species in 5 20 × 20 cm subplots. The lines show the linear model and segmented linear models which provided the best fit for the data, and dashed lines show nonsignificant linear relationship

For species evenness, segmented models with one break point had the highest empirical support for the northern and southern sites, whereas a segmented model with two breakpoints had the highest support for the central site (Table 2). At the northern site, there was no significant altitudinal variation in species below 1,228 (±18.83) m.a.s.l. (*β* = −3.13 × 10^–4^, *SE* = 3.26 × 10^–4^, *p* = .337). Above that altitude, there was a significant decline (*β* = −1.23 × 10^–2^, *SE* = 2.41 × 10^–3^, *p* < .001, Figure 3a). Similarly, at the central site there was no altitudinal variation in species evenness below 1,051 (±44.56) m.a.s.l. (*β* = −5.04 × 10^–4^, *SE* = 8.17 × 10^–4^, *p* = .538). Then, there was a significant altitudinal decline between 1,051 and 1,357 (±60.68) m.a.s.l. (*β* = −4.01 × 10^–3^, *SE* = 6.18 × 10^–4^, *p* < .001), with no significant altitudinal variation above 1,357 m.a.s.l. (*β* = −5.71 × 10^–4^, *SE* = 1.83 × 10^–3^, *p* = .755, Figure 3b). At the southern site, there was a significant altitudinal increase in species evenness to 1,174 ± 27.57 m.a.s.l. (*β* = 1.84 × 10^–3^, *SE* = 9.04 × 10^–4^, *p* = .041). After this altitude, there was a significant altitudinal declined (*β* = −3.90 × 10^–3^, *SE* = 3.69 × 10^–4^, *p* < .001, Figure 3c).

**FIGURE 4:**
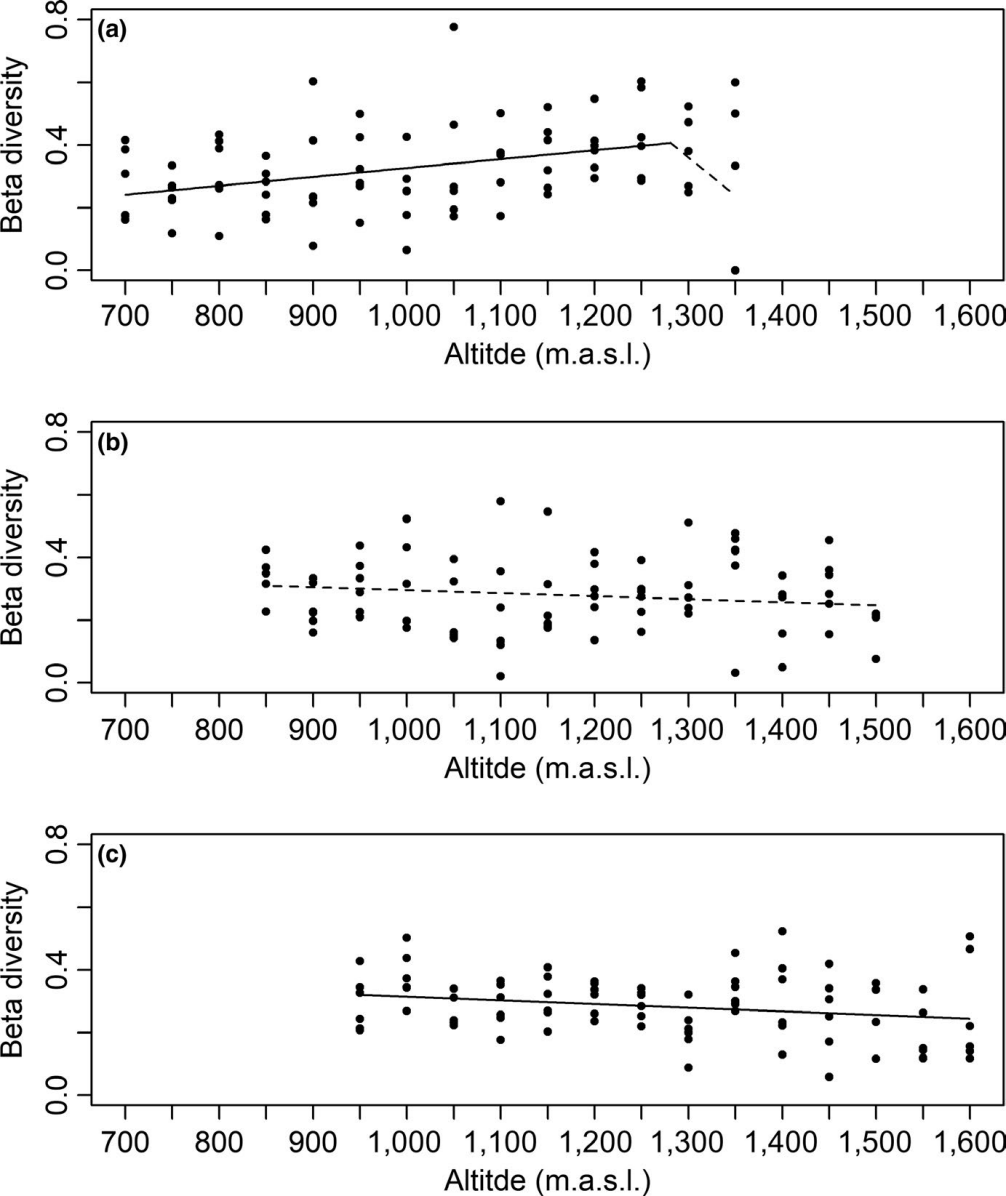
Relationships between vascular plant beta diversity and altitude at three study sites along the Swedish mountains: (a) Kebnetjokka, a northern site, (b) Hemavan, a central site, and (c) Helags, a southern site. Each point shows the distance of 1 m^2^ plots to the centroids of respective altitude in a multidimensional space formed by the present plant species. The distances were based on binary presence and absence of individual species within plots. The lines show the linear model and segmented linear models which provided the best fit for the data, and dashed lines show nonsignificant linear relationship

For beta diversity, a segmented model with one breakpoint had the highest empirical support for the northern site while unsegmented models had the highest support for the central and southern sites (Table 2). At the northern site, there was a significant altitudinal increase in beta diversity to 1,281 (± 32.53) m.a.s.l. (*β* = 2.84 × 10^–4^, *SE* = 9.52E‐05, *p* = .003). At higher altitudes, there was no significant altitudinal variation in beta diversity (*β* = −2.42E‐03, *SE* = 1.61 × 10^–3^, *p* = .132, Figure 4a). There was no significant altitudinal variation in beta diversity at the central site (*β* = −9.58 × 10^–5^, *SE* = 6.73 × 10^–5^, *p* = .158, Figure 4b), but a consistent altitudinal decline at the southern site (*β* = −1.18 × 10^–4^, *SE* = 5.36 × 10^–5^, *p* = .030, Figure 4c).

**FIGURE 5:**
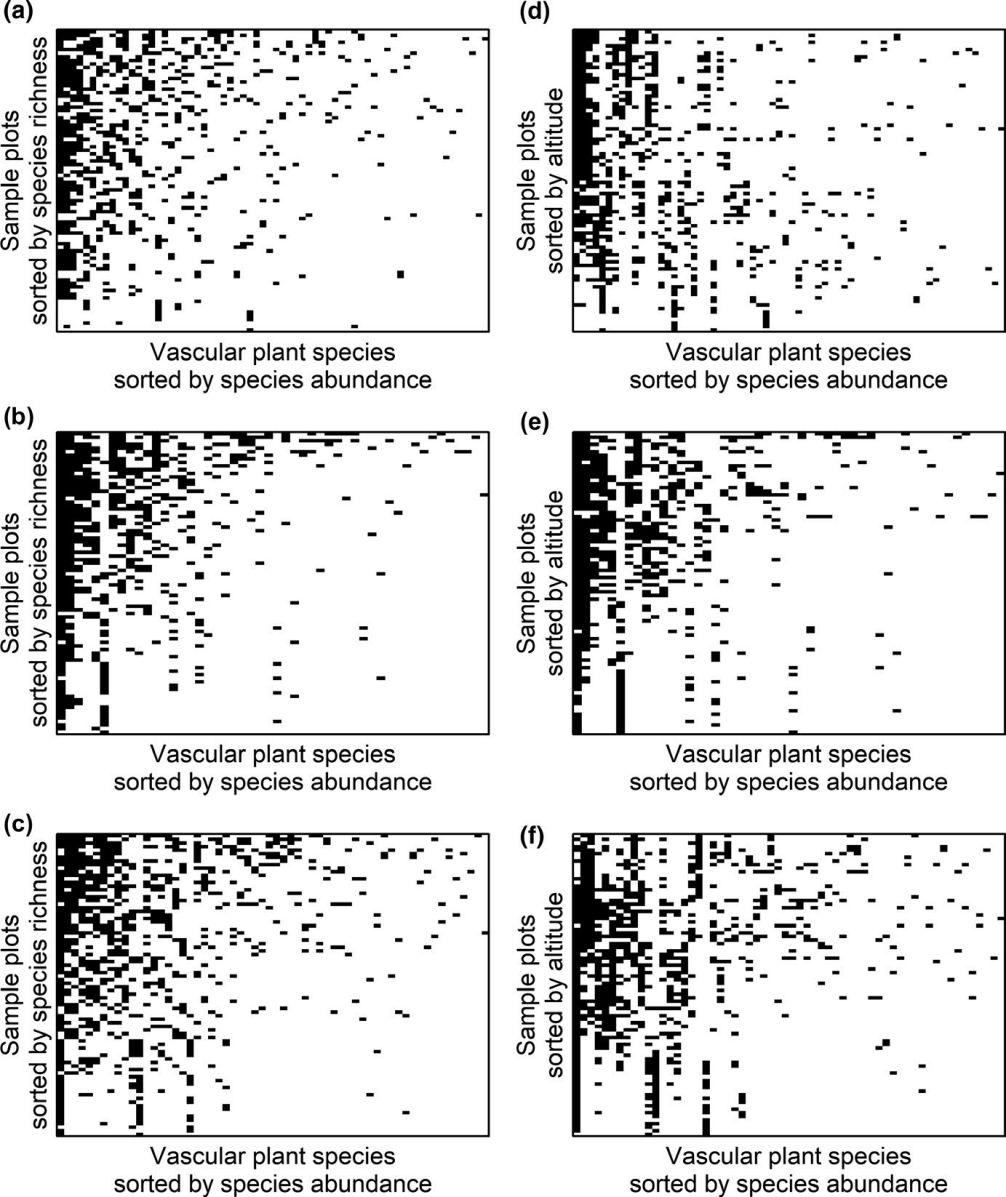
Images of binary matrices visualizing the presence or absence of plant species (columns) in 1 m^2^ plots (rows) at three study sites along the Swedish mountains: a northern site at Kebnetjokka (a, d), a central site at Hemavan (b, e), and a southern site at Helags (c, f). The plots were either sorted by species richness (a–c), generating “optimal” nestedness, or by altitude (d–f). For all matrices, the plant species were sorted by the number of plots they occurred in

**FIGURE 6:**
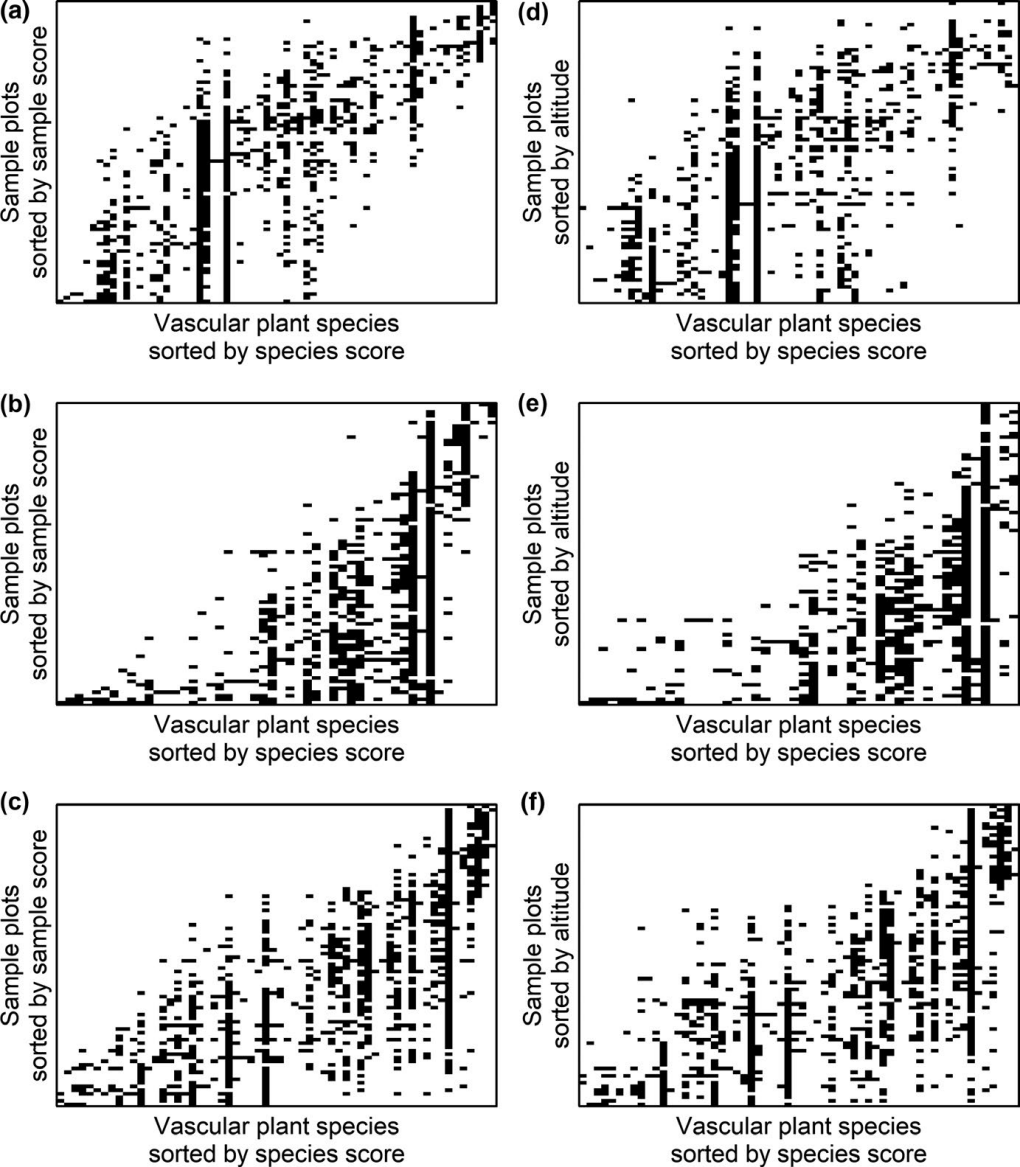
Images of binary matrices visualizing the presence or absence of plant species (columns) in 1 m^2^ plots (rows) at three study sites along the Swedish mountains: a northern site at Kebnetjokka (a, d), a central site at Hemavan (b, e), and a southern site at Helags (c, f). The plots were either sorted by reciprocal averaging scores (a–c), generating “optimal” modularity, or by altitude (d–f). For all matrices, the plant species were sorted by reciprocal averaging scores

Sorting plots by species richness generated a more spatially nested structure of community composition than expected by chance (Table 3, Figure 5a–c). However, none of the sites showed a nested pattern if the plots were sorted by altitude (Figure 5d–f), in which case all sites instead had a less nested structure than expected by chance (Table 3). All sites had more modular spatial structures of plant community composition than expected by chance (Table 3), both when the plots were sorted by reciprocal averaging scores (Figure 6a–c) and by altitude (Figure 6d–f). Sorting plots by reciprocal averaging generated 11, 12, and 11 modules at the northern, central, and southern sites, and sorting plots by altitude generated 13 modules for the northern site and 11 modules for the central and southern sites (Table 4).

**TABLE 3:** Observed and expected, under random expectations, values of the NODF index of spatial nestedness for vascular plant community composition at three sites along the Swedish mountains, for matrices where plots were ordered by species richness, generating optimal nestedness, and by altitude

Site	Plot order	NODF obs	NODF exp	*Z*	*p*
Kebnetjokka, northern site	Species richness	31.72	28.60	4.45	<.001
	Altitude	18.65	28.60	−14.16	<.001
Hemavan, central site	Species richness	46.66	36.14	9.59	<.001
	Altitude	15.44	36.14	−18.87	<.001
Helags, southern site	Species richness	38.48	31.16	10.39	<.001
	Altitude	17.76	31.16	−19.00	<.001

Note

The expected values were calculated using 1,000 randomizations and a null model that preserved species frequencies. The NODF index can take values from 0, indicating no nestedness, to 100, indicating perfect nestedness.

**TABLE 4:** Observed and expected, under random expectations, values of Barber's *Q* index of spatial modularity for vascular plant community composition at three sites along the Swedish mountains, as well as the number of identified modules, for matrices where plots were ordered by reciprocal averaging, generating optimal modularity, and by altitude

Site	Plot order	Modules	*Q* obs	*Q* exp	*Z*	*p*
Kebnetjokka, northern site	Reciprocal averaging	11	0.312	0.208	−11.21	<.001
	Altitude	13	0.313	0.208	−13.04	<.001
Hemavan, central site	Reciprocal averaging	12	0.288	0.202	−8.59	<.001
	Altitude	11	0.291	0.202	−8.80	<.001
Helags, southern site	Reciprocal averaging	11	0.301	0.195	−11.69	<.001
	Altitude	11	0.301	0.195	−11.69	<.001

Note

The expected values were calculated using 1,000 randomizations and a null model that preserved species frequencies The Q index varies from 0, indicating no modularity, to 1, indicating perfect modularity.

## 4 DISCUSSION

In line with our first expectation, we observed an expected overall decline in alpha diversity, measured both as species richness and evenness, between the lowest and highest altitude at each site. However, our second expectation, an equivalent altitudinal decline in beta diversity, was only supported by data from the southern site. Instead, we observed an altitudinal increase or no significant altitudinal variation in beta diversity at the northern and central sites, respectively. These observations suggest that the overall altitudinal decline in species richness did not result in a spatial homogenization of community composition. The patterns of altitudinal variation in alpha diversity did not vary predictably according to latitude‐related primary productivity (e.g., Currie, 1991). We also observed spatial community structures that were nested according to other environmental characteristics than altitude. Similar findings have been reported both for vascular plants (Jacquemyn, Honnay, & Pailler, 2007; Naud et al., 2019) and arthropods (Dalerum, Retief, Havemann, Chimimba, & Janse van Rensburg, 2019; Dalerum et al., 2017), and we suggest that spatial variation of species communities in a given location and area likely are formed by complex interactions of processes across several spatial and temporal scales (Willig, Kaufman, & Stevens, 2003).

Our results suggest that local characteristics, such as bedrock and moisture regimes, which are related neither to altitude nor to latitude, determine the composition of species communities at a given location. However, the observed modular community structures along the altitudinal gradients suggest that, despite strong influences of local characteristics, altitude does appear to have some general effects on vascular plant community organization. Such altitude‐related species turnover have been found previously (e.g., Naud et al., 2019), and have mainly been attributed environmental regulation of local species communities, combined with evolutionary restrictions in the regional species pool (Laiolo, Pato, & Obeso, 2018). We suggest that site‐specific variation in abiotic factors such as bedrock, local hydrology, and microtopography (Nilsson, 1991; Rydin et al., 1999), combined with local variation in biotic factors such as herbivory (Moen, 2008; Moen & Danell, 2003), interacts with the general altitudinal and latitudinal gradients in net available energy in shaping species communities across the observed landscapes. One such abiotic factor could be the presence of calcareous rocks in the northern area, leading to locally favorable botanical abiotic conditions (which also is supported by species findings in the area), disrupting both altitudinal and latitudinal patterns.

Our results were largely confirmatory with regards to alpha diversity, with observations of a unimodal altitudinal pattern (southern site), a monotonic altitudinal decline (central site), and a monotonic decline, but not along the whole gradient (northern site). Unimodal patterns have previously been observed for alpine plant communities in Fennoscandian mountains (Austrheim, 2002; Bruun et al., 2006; Naud et al., 2019; Rahbek, 1995), while a monotonic response to altitude has been observed in other alpine areas (Šťastná, Klimešová, & Doležal, 2012). However, we stress that we have observed the different patterns within a single mountain range. Unimodal patterns have been attributed to a productivity‐driven increase in competition and subsequent competitive exclusion at low altitudes, and a corresponding productivity‐driven environmental filtering at high altitudes (Callaway et al., 2002; Klanderud & Birks, 2003; Wilson & Nilsson, 2009). This explanatory model suggests that we should have found the strongest unimodal pattern at the southern site, where productivity is highest, and the weakest at the northernmost site. Our observations only partly supported these predictions, which lend further support for the importance of local characteristics in shaping local community structures.

Although we regard our results to be robust, we acknowledge several potential points of improvement for further studies. First, our study had a relatively limited sample size, with one elevation gradient per site and six replicates within each elevation level. This meant that we could not fully control for local factors such as soil humidity and microtopography. Additionally, the low number of sites along the latitudinal gradient and the relatively limited latitudinal extent could limit the generalizability of specific patterns to other areas. Second, local weather conditions often differ substantially between years, potentially leading to temporal variation in species distribution and also in identification success (Jonas, Rixen, Sturm, & Stoeckli, 2008; Walker, Webber, Arnold, & Ebert‐May, 1994). Subsequently, the time of sampling could also affect the observed plant community composition. Therefore, data series spanning several years would improve the robustness of the described patterns of community composition within the altitudinal gradients. Third, our sampling design did not allow any evaluations of altitudinal and latitudinal effects on plant communities across several spatial scales. Since scale dependence previously has been suggested as important when quantifying diversity along environmental gradients (Dalerum et al., 2019; Naud et al., 2019; Rahbek, 2005), we suggest that future studies adopt experimental designs that allow for evaluations across different spatial scales. Despite these limitations, this study is one of the first studies in this region to investigate the alpine vegetation patterns on several mountains across a latitudinal span in one single year, adding information to the yet small body of knowledge on alpine vegetation patterns in Scandinavia.

## 5 CONCLUSIONS

In conclusion, the patterns of species richness, species evenness, and beta diversity, as well as spatially nested community structures that did not follow altitudinal gradients, point to strong effects of local characteristics for site‐specific community composition. However, we found consistent spatial modularity along the investigated altitudinal gradients, which support that altitude do impose limits to distribution ranges of individual species, and imply that such limits are at least to some extent governed by abiotic regulation. We suggest that spatial variation of vascular communities along altitudinal and latitudinal gradients is likely caused by a combination of interactions of processes that occur across spatial scales. We propose that further studies focus at identifying interactions of biotic and abiotic processes, and at which scales these processes are ecologically relevant. We stress the importance of detailed information of plant diversity patterns and community structure, particularly in areas such as the Swedish mountains, since such information is paramount for our ability to investigate ecological consequences of climate‐induced range shifts.

## CONFLICT OF INTEREST

The authors declare no conflict of interest.

## AUTHOR CONTRIBUTION

**Johannes Måsviken:** Conceptualization (equal); data curation (equal); formal analysis (equal); funding acquisition (equal); investigation (lead); methodology (equal); project administration (supporting); software (supporting); validation (equal); visualization (equal); writing – original draft (lead); writing – review and editing (lead). **Fredrik Dalerum:** Conceptualization (equal); data curation (equal); formal analysis (equal); funding acquisition (lead); investigation (supporting); methodology (equal); project administration (lead); software (lead); supervision (equal); validation (equal); visualization (supporting); writing – original draft (supporting); writing – review and editing (supporting). **Sara Cousins:** Conceptualization (supporting); investigation (supporting); methodology (equal); project administration (supporting); supervision (equal); validation (equal); writing – original draft (supporting); writing – review and editing (supporting). 

## ETHICAL APPROVAL

The study is in compliance with ethical standards.

## Data Availability

Data on species presences, Shannon evenness, and centroid distances used for estimation of beta diversity, as well as binary presence–absence matrices, are available from datadryad (https://datadryad.org/: https://doi.org/10.5061/dryad.0p2ngf1wq).

## APPENDIX 1

**TABLE A1:** List of species and taxa of vascular plants observed at the northern site Kebnetjokka, including the number of transects (i.e., elevation strata) and the number of plots each species or taxa were observed in

Species/Taxa	No transects	No plots
*Agrostis mertensii*	1	1
*Andromeda polifolia*	1	2
*Antennaria alpina*	4	8
*Antennaria dioica*	3	7
*Antennaria* sp.	8	17
*Arctostaphylos uva‐ursi*	6	20
*Arctous alpina*	10	24
*Astragalus alpinus*	4	6
*Bartsia alpina*	3	4
*Betula nana*	7	24
*Bistorta vivipara*	10	17
*Campanula uniflora*	1	1
*Cardamine bellidifolia*	3	7
*Carex vaginata*	3	4
*Carex bigelowii*	5	7
*Carex rupestris*	3	5
*Carex* sp.	10	22
*Chamaenerion angustifolium*	2	2
*Cornus suecica*	1	2
*Diapensia lapponica*	2	2
*Dryas octopetala*	6	13
*Empetrum nigrum* subsp. *hermaphroditum*	12	59
*Equisetum pratense*	1	1
*Erigeron* sp.	3	3
*Erigeron uniflorus*	3	3
*Euphrasia wettsteinii*	2	2
*Gymnocarpium dryopteris*	1	1
*Harrimanella hypnoides*	5	11
*Hieracium* sp.	9	22
*Huperzia selago* subsp. *arctica*	1	2
*Juncus* sp.	1	1
*Juncus trifidus*	10	31
*Juniperus communis*	7	12
*Kalmia procumbens*	4	5
*Linnaea borealis*	7	20
*Luzula arcuata*	4	15
*Luzula* sp.	4	4
*Luzula spicata*	3	3
*Lycopodium alpinum*	2	2
*Lycopodium annotinum*	2	3
*Lysimachia europaea*	4	12
*Pedicularis lapponica*	4	9
*Pedicularis* sp.	3	3
*Phyllodoce caerulea*	7	14
*Pinguicula vulgaris*	1	1
*Poa alpina*	12	39
Poaceae spp. 1	12	26
Poaceae spp. 2	1	1
*Potentilla crantzii*	1	1
*Ranunculus glacialis*	3	7
*Rhodiola rosea*	1	1
*Salix herbacea*	9	33
*Salix lapponum/glauca*	2	3
*Saussurea alpina*	3	3
*Sibbaldia procumbens*	3	4
*Silene acaulis*	6	13
*Solidago virgaurea*	6	9
*Taraxacum* sp.	1	1
*Thalictrum alpinum*	4	4
*Trisetum spicatum*	2	5
Unknown (Ranunculaceae?)	1	1
*Vaccinium myrtillus*	6	10
*Vaccinium uliginosum*	12	47
*Vaccinium vitis‐idaea*	13	63
*Veronica alpina*	1	2
*Viola biflora*	6	7

**TABLE A2:** List of species and taxa of vascular plants observed at the central site Hemavan, including the number of transects (i.e., elevation strata) and the number of plots each species or taxa were observed in

Species/Taxa	No transects	No plots
*Alchemilla alpina*	3	5
*Antennaria dioica*	6	3
*Antennaria* sp.	3	9
*Anthoxanthum nipponicum*	1	4
*Arctous alpina*	2	1
*Astragalus alpinus*	4	2
*Bartsia alpina*	9	6
*Bistorta vivipara*	1	28
*Calamagrostis* sp.	1	1
*Campanula uniflora*	4	1
*Cardamine bellidifolia*	6	5
*Carex bigelowii*	9	15
*Carex* sp.	1	22
*Cerastium* sp.	5	2
*Diapensia lapponica*	1	6
*Dryas octopetala*	8	1
*Empetrum nigrum* subsp. *hermaphroditum*	1	33
*Equisetum arvense* subsp. *alpestre*	1	1
*Equisetum* sp.	9	1
*Harrimanella hypnoides*	9	30
*Hieracium* sp.	6	20
*Huperzia selago* subsp. *arctica*	12	11
*Juncus trifidus*	5	62
*Kalmia procumbens*	9	12
*Luzula arcuata*	2	29
*Luzula* sp.	2	2
*Lycopodium alpinum*	4	2
*Lysimachia europaea*	7	7
*Pedicularis lapponica*	8	13
*Phyllodoce caerulea*	5	24
*Poa alpina*	9	7
Poaceae spp.	2	15
*Potentilla crantzii*	2	3
*Ranunculus acris*	1	1
*Ranunculus glacialis*	1	2
*Rumex acetosa* subsp. *lapponicus*	14	3
*Salix herbacea*	1	79
*Salix reticulata*	2	1
*Saussurea alpina*	3	3
*Sibbaldia procumbens*	3	7
*Silene acaulis*	2	4
*Solidago virgaurea*	1	3
*Taraxacum* sp.	1	2
*Thalictrum alpinum*	1	1
*Tofieldia pusilla*	7	1
Unknown (Gentianaceae/*Campanula*?)	1	1
*Vaccinium myrtillus*	8	26
*Vaccinium uliginosum*	10	21
*Vaccinium vitis‐idaea*	3	37
*Viola biflora*	1	7

**TABLE A3:** List of species and taxa of vascular plants observed at the southern site Helags, including the number of transects (i.e., elevation strata) and the number of plots each species or taxa were observed in

Species/Taxa	No transects	No plots
*Alchemilla alpina*	6	19
*Andromeda polifolia*	1	1
*Antennaria dioica*	12	39
*Antennaria* sp.	2	3
*Anthoxanthum nipponicum*	3	3
*Arctostaphylos uva‐ursi*	1	2
*Arctous alpina*	2	2
Asteraceae sp.	4	4
*Astragalus alpinus*	3	5
*Bartsia alpina*	4	4
*Betula nana*	4	18
*Bistorta vivipara*	11	32
*Calluna vulgaris*	2	7
*Cardamine bellidifolia*	2	2
*Carex bigelowii/nigra*	6	11
*Carex* sp.	11	30
*Cerastium* sp.	3	3
*Diapensia lapponica*	2	4
*Dryas octopetala*	3	8
*Empetrum nigrum* subsp. *hermaphroditum*	10	45
*Erigeron* sp.	3	5
*Galium boreale*	2	2
*Harrimanella hypnoides*	9	20
*Hieracium* sp.	8	35
*Huperzia selago* subsp. *arctica*	6	9
*Juncus* sp.	4	9
*Juncus trifidus*	10	37
*Juniperus communis*	3	6
*Kalmia procumbens*	9	30
*Luzula arcutata/spicata*	2	3
*Luzula* sp.	6	23
*Lycopodium alpinum*	7	13
*Lysimachia europaea*	4	7
*Pedicularis lapponica*	4	5
*Pedicularis* sp.	2	2
*Phyllodoce caerulea*	9	22
*Pinguicula vulgaris*	1	2
*Poa alpina*	8	19
Poaceae spp. *1*	12	25
Poaceae spp. *2*	3	3
*Potentilla crantzii*	3	3
*Pyrola* sp.	2	2
*Ranunculus glacialis*	4	16
*Ranunculus acris*	1	1
*Rumex acetosa* subsp. *lapponicus*	2	2
*Salix herbacea*	14	73
*Salix reticulata*	2	2
*Saussurea alpina*	5	7
*Saxifraga oppositifolia*	1	1
*Sibbaldia procumbens*	9	22
*Silene acaulis*	7	11
*Scorzoneroides autumnalis* var. *pratensis*	2	2
*Thalictrum alpinum*	4	6
*Tofieldia pusilla*	2	4
Unknown	2	2
Unknown (Gentianaceae/*Campanula*?)	5	8
*Vaccinium myrtillus*	8	28
*Vaccinium uliginosum*	7	16
*Vaccinium vitis‐idaea*	9	40
*Viola biflora*	4	7
